# Enzyme-cascade-amplified colorimetric biosensing platform for sub-nanomolar methylmercury in environmental waters

**DOI:** 10.1039/d5ra09313a

**Published:** 2026-01-07

**Authors:** Wanyan Wu, Yan Guo, Lidan Deng, Wenwu Gong, Shunyu Hu, Fen Liu, Chang-ye Hui

**Affiliations:** a Department of Toxicology, School of Public Health, Southern Medical University Guangzhou China; b Shenzhen Prevention and Treatment Center for Occupational Diseases Shenzhen China hcy_sypu@hotmail.com; c School of Public Health, Southern Medical University Guangzhou China

## Abstract

We developed an enzyme-cascade-amplified whole-cell biosensor for sub-nanomolar methylmercury (MeHg) detection in environmental waters. By integrating a MerB-based organomercurial lyase with a water-soluble indigoidine reporter system in a decoupled genetic circuit, we achieved a limit of detection (LOD) of 0.04 nM and a linear range of 0.02–1.22 nM. Systematic comparison of intracellular *versus* surface-displayed MerB strategies revealed that intracellular expression (TOP10/pCon-IND-C-B) provided superior sensitivity. The biosensor demonstrated robust performance across tap water, surface water, and seawater matrices (*R*^2^ > 0.98) with negligible matrix effects, enabling direct colorimetric quantification of culture supernatants. Notably, metabolic burden from pigment synthesis dominated the dose-response profile rather than MeHg cytotoxicity. This biosensor offers a cost-effective alternative for screening MeHg in environmental water, with detection limits that far exceed WHO drinking water guidelines and a potential for high-throughput environmental monitoring.

## Abstract 2

An enzyme-cascade-amplified whole-cell biosensor detects methylmercury in water with an LOD of 0.04 nM *via* indigoidine pigment production. The platform enables cost-effective, high-throughput screening for environmental monitoring.

## Graphical abstract

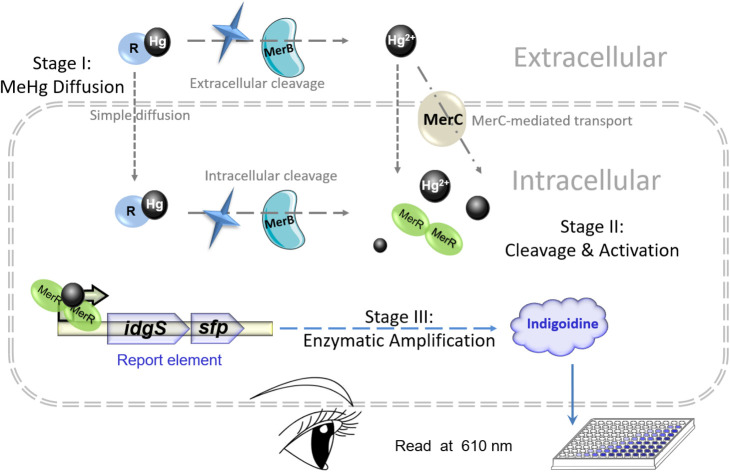

## Introduction

Mercury (Hg) is a globally distributed contaminant that cycles between atmospheric, terrestrial, and aquatic reservoirs through complex biogeochemical processes.^1^ In aquatic environments, inorganic Hg(ii) is microbially converted to methylmercury (MeHg), a neurotoxic organomercurial that bioaccumulates and biomagnifies through food webs, posing severe risks to human health and ecosystems.^2^ MeHg concentrations in water bodies are typically regulated at nanomolar levels (*e.g.*, the WHO drinking water guideline is approximately 30 nM).^3^ However, sensitive monitoring tools capable of detecting sub-nanomolar concentrations in diverse environmental matrices, including tap water, surface water, and seawater, remain critically needed.^3,4^

Current methods for mercury speciation rely heavily on instrumental techniques such as liquid chromatography–inductively coupled plasma mass spectrometry.^5,6^ While sensitive, these approaches require expensive instrumentation, complex sample preparation (*e.g.*, extraction, derivatization, separation), and specialized expertise, which limits their applicability for frequent, on-site environmental screening. In response, biosensors have emerged as promising alternatives, offering rapid, cost-effective, and field-deployable detection.^7^ Whole-cell biosensors, particularly those engineered around the natural *mer* operon, have been extensively explored for Hg(ii) sensing.^8^ However, conventional reporters, such as fluorescence and bioluminescence, suffer from high detection thresholds, a lack of visual readout, and limited sensitivity for ultratrace analysis.^9–11^ Pigment-based reporting systems address these limitations through two key advantages: (i) enzymatic pigment synthesis provides cascade amplification, enhancing sensitivity; and (ii) colorimetric detection *via* visible-light spectrophotometry eliminates the need for sophisticated instrumentation while enabling high-throughput screening.^12,13^ Water-soluble pigments, such as the blue pigment indigoidine, further simplify analysis by allowing for the direct quantification of cell-free supernatants without the need for organic extraction.^14–16^

The *mer* operon encodes several proteins critical for mercury detoxification, including the organomercurial lyase MerB, which cleaves the carbon–Hg bond in MeHg to release Hg(ii).^17^ Since the *mer* regulator MerR specifically responds to Hg(ii), but not MeHg, the incorporation of MerB into biosensor circuits enables the indirect detection of organomercury species through this conversion step.^3^ Previous studies have leveraged MerA (mercuric reductase) and MerB to expand the detection spectrum and improve bioremediation capacity.^18,19^ Nevertheless, the integration of these modules with strong, water-soluble pigment reporters to enable convenient and effective detection remains inadequately explored.

Herein, we report an enzyme-cascade-amplified whole-cell biosensor that merges MerB-based MeHg cleavage with indigoidine synthesis for sub-nanomolar colorimetric detection. By optimizing a decoupled genetic circuit and incorporating the Hg(ii) transporter MerC, we achieved a limit of detection (LOD) of 0.04 nM and a quantitative range of 0.02–1.22 nM. Critically, we systematically compared intracellular *versus* surface-displayed MerB strategies, revealing that intracellular expression provides superior sensitivity. The biosensor demonstrated negligible matrix effects in tap water, surface water, and seawater, validating its potential for practical environmental monitoring. This work advances the development of low-threshold tools for MeHg screening and highlights the importance of metabolic matching in engineering high-performance whole-cell biosensors.

## Materials and methods

### Bacterial strains, plasmids, and reagents

Bacterial strains and plasmids used in this study are listed in Table 1. *E. coli* strains harboring recombinant plasmids were cultured in LB medium containing 50 µg mL^−1^ ampicillin (5 g L^−1^ yeast extract, 10 g L^−1^ tryptone, 10 g L^−1^ NaCl) at 30 °C with shaking at 150 rpm. Methylmercury (MeHg) stock solutions were freshly prepared using analytical-grade methanol (Aladdin, Shanghai, China). Oligonucleotide primers and synthetic DNA fragments were synthesized by Sangon Biotech (Shanghai, China). Sequences of key genetic components are detailed in Table S1.

**Table 1:** Bacterial strains and genetic constructs used in this study

Strains and plasmids	Genotypes or description	Ref.
*E. coli* strain		
TOP10	F^−^*Φ*80 *lac*ZΔM15 Δ*lac*X74 *rec*A1	Invitrogen
Vectors		
pCon-IND	pHg-IdgS-Sfp derivative with a constitutive P_*ceuR*_ promoter inserted in front of the *merR* gene	21
pCon-IND-C	pCon-IND derivative containing a constitutive *P*_J23119_-controlled *merC* gene inserted as a *Sac*I-*Hin*dIII fragment	21
pCon-IND-C-B	pCon-IND-C derivative containing a constitutive P637-controlled merB gene inserted as a *Hin*dIII-*Xho*I fragment	This study
pCon-IND-C-surB	pCon-IND-C derivative containing a constitutive P637-controlled inaPb-merB gene inserted as a *Hin*dIII-*Xho*I fragment	This study

Safety Warning: methylmercury (MeHg) is an extremely potent neurotoxin that can be absorbed through inhalation, dermal contact, or ingestion.^20^ All manipulations must be performed in a fume hood, and personnel are required to wear nitrile gloves, safety goggles, and a lab coat. Waste solutions containing MeHg must be treated with sodium sulfide to generate HgS precipitate prior to disposal as hazardous waste. All experiments in this study were conducted in a laboratory meeting Biosafety Level 2 (BSL-2) standards.

### Plasmid construction

The plasmid pCon-IND, a previously reported Hg(ii) biosensing plasmid, served as the parental construct.^21^ It contains an *Nde*I-*Sac*I fragment that encodes the reporter genes *idgS* and *sfp*, which are a non-ribosomal peptide synthetase from *Streptomyces* (GenBank: ARE61062.1) and a phosphopantetheinyl transferase from *Bacillus* (GenBank: ABW74629.1), respectively. Additionally, a *Bgl*II-*Xba*I fragment harbors a decoupled Hg(ii)-responsive element comprising a constitutive promoter upstream of *merR* and a divergent P*mer* promoter.

To generate pCon-IND-C, a constitutive *P*_J23119_-controlled *merC* gene (encoding the Hg(ii) transporter MerC) was inserted downstream of the reporter cassette as a *Sac*I-*Hin*dIII fragment.

For methylmercury sensing, two MerB expression modules were constructed at the *Hin*dIII-*Xho*I site downstream of the *merC* cassette: (i) an intracellularly expressed MerB under the constitutive P637 promoter (pCon-IND-C-B), and (ii) a surface-displayed MerB fused with the ice nucleation protein InaPb (pCon-IND-C-surB).

All biosensing plasmids were transformed into *E. coli* TOP10 *via* chemical transformation to generate whole-cell biosensors for subsequent characterization.

### Comparison of biosensing performance

To evaluate the sensitivity and dynamic range of the three biosensors toward MeHg, freshly prepared overnight cultures of TOP10/pCon-IND-C, TOP10/pCon-IND-C-B, and TOP10/pCon-IND-C-surB were inoculated at 2% (v/v) into fresh LB medium containing MeHg at final concentrations of 0, 0.02, 0.04, 0.08, 0.15, 0.31, 0.61, 1.22, 2.44, 4.88, 9.77, 19.53, 39.06, 78.13, 156.25, 312.5, 625, 1250, 2500, and 5000 nM (two-fold serial dilution). Following incubation at 30 °C with shaking at 150 rpm for 4 h, bacterial growth and pigment signals were quantified.

### Validation in environmental water matrices

Environmental water samples were collected from the following sources: laboratory-purified deionized water, municipal tap water, surface water from Donghu Park (Luohu District, Shenzhen, Guangdong Province, China), and seawater from Daya Bay (Yantian District, Shenzhen, Guangdong Province, China). Samples were clarified by centrifugation at 10 000 rpm for 10 min, and the supernatants were filtered through a 0.22 µm membrane filter (Millipore, Billerica, MA, USA) to remove particulates and microorganisms.

We prepared sensing culture systems by mixing four types of water with 10× LB broth, 10× NaCl-free LB broth, and sterile water in specific volume ratios, as previously described in Table 2.^22,23^

**Table 2:** Biosensing culture system for various environmental water samples

Component	Deionized water	Tap water	Surface water	Seawater
Water samples	90%	90%	90%	50%
10× LB	10%	10%	10%	—
10× NaCl-free LB	—	—	—	10%
Sterile water	—	—	—	40%

Overnight cultures of TOP10/pCon-IND-C-B were inoculated at a 2% (v/v) concentration into four sensing-culture systems, each supplemented with 50 µg mL^−1^ of ampicillin. MeHg was introduced through a two-fold serial dilution to achieve final concentrations of 0, 0.02, 0.03, 0.06, 0.13, 0.25, 0.5, and 1 nM. Following incubation at 30 °C with shaking at 150 rpm for 4 hours, we measured bacterial density and pigment signals.

### Measurement of bacterial density and pigment signal

Aliquots (100 µL) of induced culture were transferred to a 96-well microplate, and the optical density at 600 nm (OD_600_) was measured using a microplate reader (BioTek Epoch, Winooski, VT, USA). After centrifugation at 8000 g for 2 min (MiniSpin centrifuge, Eppendorf, Hauppauge, NY, USA), 100 µL aliquots of the supernatant containing water-soluble indigoidine were transferred to a fresh microplate and quantified at 610 nm. The absorbance at 610 nm (*A*_610_) was recorded as the indigoidine signal.^21^ Bacterial cell density was calculated by subtracting the OD_600_ of the cell-free supernatant from the total culture OD_600_.^14,16^

### Statistical analysis

Statistical analysis was performed using IBM SPSS 26.0. Data fitting and graphical visualization were performed using OriginPro 2024. Data are presented as mean ± standard deviation (mean ± SD, *n* ≥ 3). Comparisons between two groups were performed using student's *t*-test. Comparisons involving three or more groups were performed using one-way analysis of variance (ANOVA). Unless otherwise stated, differences were considered significant at *p* < 0.05. The limit of detection (LOD) was calculated according to the Clinical and Laboratory Standards Institute (CLSI) EP17-A guideline: LOD = LOB + 1.645 × SD of low-concentration samples, where the limit of blank (LOB) is defined as the mean blank value plus 1.645 times the standard deviation of the blank values.^13^

## Results and discussion

### Evaluation of MeHg sensing performance of three biosensors

**Fig. 1:**
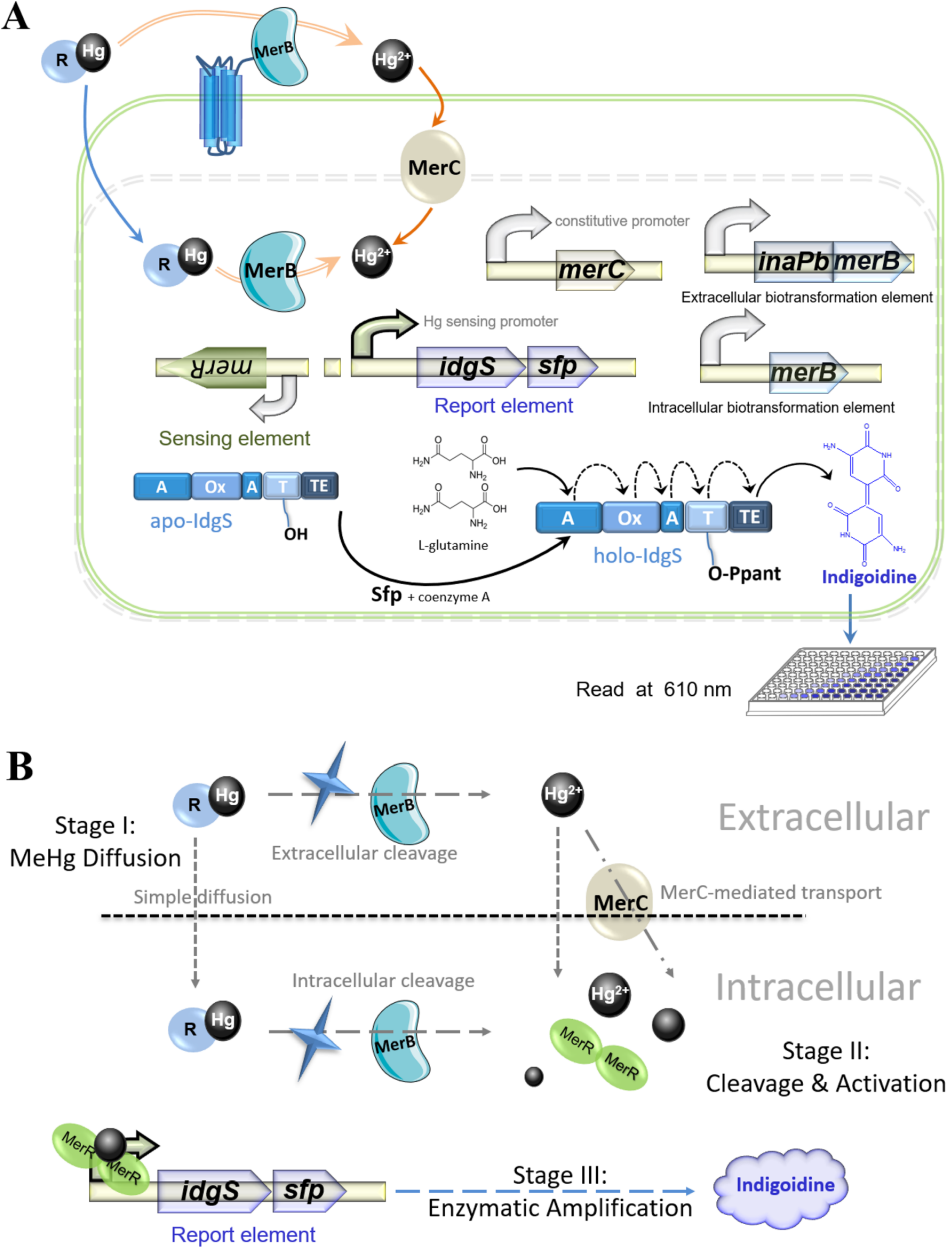
(A) Schematic of the organomercury biosensing strategy comprising: (i) a decoupled Hg(ii)-responsive module, (ii) an indigoidine reporter module, (iii) a Hg(ii) transmembrane transport module (MerC), and (iv) modules for intracellular and extracellular organomercury cleavage. (B) Schematic illustration of the MeHg sensing process in three sequential stages: (i) diffusion of MeHg into the system, (ii) cleavage/activation events, and (iii) enzymatic signal generation/readout.

The biosensing strategy employed in this study is illustrated in Fig. 1. A decoupled genetic circuit was engineered based on our previous design, incorporating the *idgS-sfp* gene cluster as the reporting module. In this architecture, the Hg(ii)-responsive regulator MerR is constitutively expressed from the P*ceuR* promoter positioned upstream of a divergent P*mer* promoter. The P*mer* promoter drives expression of three functional modules: (i) the *idgS-sfp* reporter operon, (ii) a MerB-based organomercurial lyase module for MeHg cleavage, and (iii) the *merC*-encoded Hg(ii) transporter to enhance Hg(ii) influx. Constitutive promoters also control the latter two modules to ensure stable expression.

The sensing principle operates as follows: in the absence of Hg(ii), dimeric MerR binds to P*mer* and represses transcription of the downstream genes.^24^ Upon exposure to MeHg, either intracellularly expressed or surface-displayed MerB catalyzes the cleavage of MeHg to Hg(ii), which binds MerR and converts it into a transcriptional activator, which activates P*mer*, initiating transcription of the *idgS-sfp* cluster. The expressed Sfp phosphopantetheinyl transferase activates apo-IdgS to holo-IdgS, which catalyzes the condensation of two l-glutamine molecules to produce the water-soluble blue pigment indigoidine.^25^ This pigment exhibits a characteristic absorption maximum at 610 nm, enabling colorimetric quantification.^21^

We hypothesized that anchoring InaPb-MerB fusion protein on the outer membrane would facilitate extracellular cleavage of MeHg, thereby preventing the cytotoxicity associated with intracellular accumulation of MeHg and enabling detection at elevated concentrations.

### MeHg sensing by the organomercury lyase-free biosensor TOP10/pCon-IND-C

**Fig. 2:**
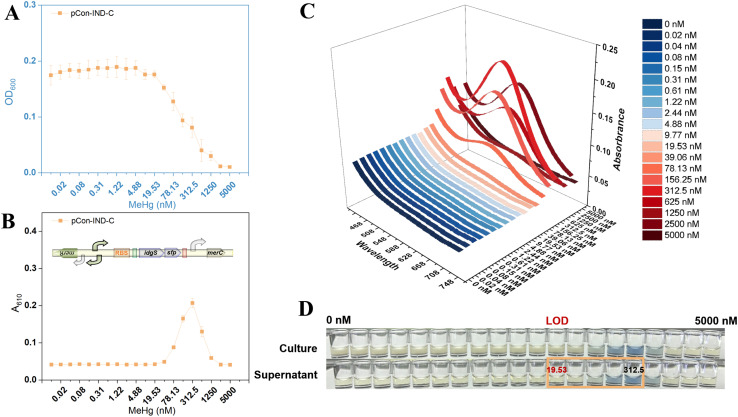
MeHg dose-response of TOP10/pCon-IND-C. (A) Bacterial growth (OD_600_) after exposure to varying MeHg concentrations. (B) Changes in absorbance of culture supernatant at 610 nm as the indigoidine signal. (C) Three-dimensional visible-light scanning spectra showing absorbance (*Z*-axis) as a function of wavelength (*X*-axis, 430–750 nm) and MeHg concentration (*Y*-axis) in culture supernatants. (D) Representative images of bacterial cultures and supernatants after MeHg exposure.

The growth of TOP10/pCon-IND-C was inhibited with increasing MeHg concentrations, as evidenced by a gradual decrease in OD_600_ that became particularly pronounced at concentrations exceeding ∼19.6 nM (Fig. 2A). Over the range of 0–5000 nM, pigment production exhibited a typical unimodal dose-response curve, reaching a maximum at ∼312.5 nM followed by a sharp decline due to cytotoxicity-induced growth inhibition and pigment synthesis attenuation (Fig. 2B). Full-spectrum scanning revealed that indigoidine was secreted into the culture medium with a characteristic absorption maximum at 610 nm, which remained constant across all MeHg concentrations tested (Fig. 2C). Fig. 2D compares bacterial cultures with cell-free supernatants, demonstrating that the pigment remained exclusively in the supernatant after centrifugation.

Unlike Hg(ii), which is actively imported *via* MerT/MerP and MerC transporters, MeHg uptake does not rely on *mer* operon-encoded transport systems.^8,26^ This is likely attributed to its physicochemical properties, including a small molecular size and higher lipophilicity, which enable effective membrane permeation without the need for transporter assistance.^27^ MeHg is sufficiently lipophilic to traverse the *E. coli* membrane *via* simple diffusion, particularly when complexed with monovalent anions (*e.g.*, Cl^−^) present in LB medium.^3^

Since dimeric MerR exclusively responds to Hg(ii), the observed pigment signal upon high-concentration MeHg exposure can be rationalized as follows. The native *E. coli* TOP10 strain lacks the *merB* gene, excluding the possibility of endogenous MeHg cleavage.^3^ First, trace impurities of inorganic Hg(ii) may persist in the MeHg stock solution despite its high purity. Second, ambient light exposure during storage or incubation could induce spontaneous photodegradation of MeHg, generating trace Hg(ii).^2^ These sources of Hg(ii) likely account for the pigment response observed at concentrations greater than 312.5 nM, where cytotoxicity severely suppresses biosensor growth, leading to rapid signal attenuation and the unimodal response profile.^4^ The invariant 610 nm peak across all concentrations indicates that indigoidine maintains a stable chemical structure in the supernatant, which is advantageous for accurate colorimetric quantification.

### MeHg sensing by the intracellular MerB-expressing biosensor TOP10/pCon-IND-C-B

**Fig. 3:**
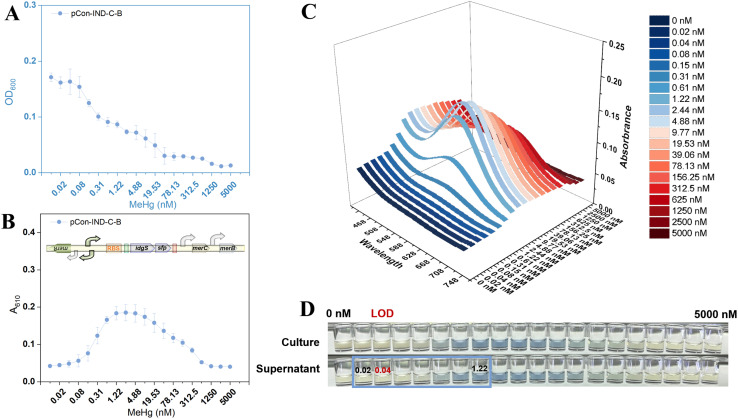
MeHg dose-response of TOP10/pCon-IND-C-B. (A) Bacterial growth (OD_600_) after exposure to varying MeHg concentrations. (B) Changes in absorbance of culture supernatant at 610 nm as the indigoidine signal. (C) Three-dimensional visible-light scanning spectra showing absorbance (*Z*-axis) as a function of wavelength (*X*-axis, 430–750 nm) and MeHg concentration (*Y*-axis) in culture supernatants. (D) Representative images of bacterial cultures and supernatants after MeHg exposure.

Increasing MeHg concentrations inhibited cell growth in a dose-dependent manner, with bacterial density (OD_600_) declining significantly above 0.08 nM and complete growth arrest observed at concentrations exceeding 19.5 nM (Fig. 3A). Fig. 3B shows the dose-response profile, which again exhibited a unimodal relationship: pigment accumulation initiated above 0.08 nM, peaked at approximately 2.44 nM, and declined gradually thereafter. Notably, bacterial density decreased continuously across the entire concentration range. The indigoidine pigment secreted by this strain maintained a characteristic absorption maximum at 610 nm that was invariant with MeHg concentration (Fig. 3C). Fig. 3D demonstrates that the pigment remained exclusively in the supernatant after centrifugation.

Compared with TOP10/pCon-IND-C, the intracellular expression of MerB in TOP10/pCon-IND-C-B shifted the pigment signal peak leftward to ∼2.44 nM, representing a two-order-of-magnitude improvement in detection limit relative to the parent sensor (peak at ∼312.5 nM). This demonstrates that intracellular MerB efficiently cleaves MeHg to Hg(ii), which subsequently activates the pigment reporter system. Unlike TOP10/pCon-IND-C, where growth inhibition at concentrations greater than 19.6 nM resulted directly from MeHg cytotoxicity, the decrease in density in TOP10/pCon-IND-C-B at concentrations as low as 0.08 nM was mainly due to the metabolic burden from indigoidine synthesis.^16^ Possibly forming a “metabolic stress-pigment attenuation” peak. The incorporation of the intracellular cleavage module lowered the LOD to 0.04 nM. This sub-nanomolar detection limit, enabled by enzymatic signal amplification, is substantially superior to previously reported whole-cell biosensors for organomercury detection.^3,4^

### MeHg sensing by the surface-displayed MerB biosensor TOP10/pCon-IND-C-surB

**Fig. 4:**
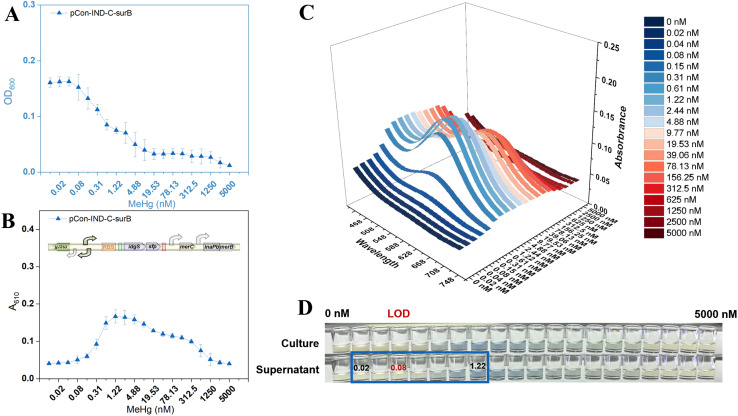
MeHg dose-response characteristics of TOP10/pCon-IND-C-surB. (A) Bacterial growth (OD_600_) after exposure to varying MeHg concentrations. (B) Indigoidine signal (*A*_610_) of culture supernatants. (C) Three-dimensional visible-light scanning spectra showing absorbance (*Z*-axis) as a function of wavelength (*X*-axis, 430–750 nm) and MeHg concentration (*Y*-axis) in culture supernatants. (D) Representative images of bacterial cultures and supernatants.

MeHg exposure inhibited sensor cell growth in a dose-dependent manner, with bacterial density (OD_600_) decreasing markedly above 0.04 nM and complete growth arrest at concentrations exceeding 9.77 nM (Fig. 4A). The dose-response relationship exhibited a unimodal profile: pigment accumulation initiated above 0.08 nM, peaked at approximately 1.22 nM, and declined gradually thereafter (Fig. 4B). Bacterial density decreased continuously across the entire concentration range. The secreted indigoidine pigment maintained a characteristic absorption maximum at 610 nm (Fig. 4C) and remained exclusively in the supernatant after centrifugation (Fig. 4D).

Unlike TOP10/pCon-IND-C-B with intracellular MerB expression, TOP10/pCon-IND-C-surB employs surface-displayed InaPb-MerB fusion proteins to catalyze extracellular MeHg cleavage.^28^ Then, the resulting Hg(ii) is transported into the cell *via* MerC to activate the pigment reporter system.^26^ However, compared with the intracellular strategy, surface display yielded lower pigment signal intensity and a modestly elevated LOD (0.08 nM *versus* 0.04 nM), failing to achieve the anticipated sensitization effect. This is likely because free diffusion of MeHg into cells provides direct access to intracellular MerB in the pCon-IND-C-B system. In contrast, exclusive reliance on surface-displayed MerB limits the amount of MeHg that can be processed, even with MerC-assisted Hg(ii) transport.

### Comparative quantitative detection ranges of the three biosensors

As shown in Fig. S1, introduction of MerB into TOP10/pCon-IND-C-B and TOP10/pCon-IND-C-surB restricted sensor growth even at extremely low MeHg concentrations, primarily due to metabolic burden from pigment synthesis. The blue coloration of culture supernatants was visually discernible at low concentrations, enabling naked-eye detection.

**Fig. 5:**
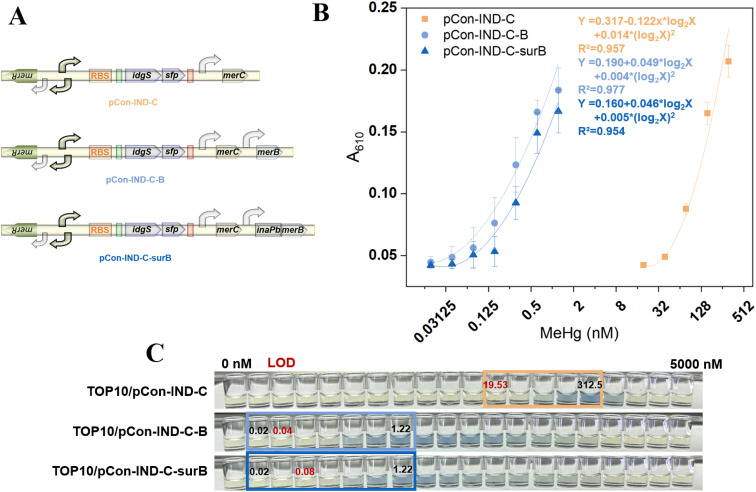
Comparative performance of the three biosensors. (A) Progressive genetic design of the plasmids. (B) Regression analysis of pigment signal (*A*_610_) *versus* MeHg concentration plotted on a log_2_ scale. (C) Representative images of culture supernatants; boxed regions indicate the quantifiable linear range, with red values indicating calculated LODs.

The progressive genetic design (Fig. 5A) enabled direct comparison of how module incorporation affected the quantitative detection range (Fig. 5B). TOP10/pCon-IND-C (lacking MerB) showed a robust nonlinear relationship in the high-concentration range of 19.53–312.5 nM (*R*^2^ = 0.957), responding to trace Hg(ii) impurities and photodegradation products. Both TOP10/pCon-IND-C-B and TOP10/pCon-IND-C-surB, equipped with intracellular and surface-displayed MerB, respectively, exhibited strong nonlinear relationships in the 0.02–1.22 nM range (*R*^2^ = 0.977 and 0.954) (Fig. 5B). Statistical significance (two-tailed *t*-test, *P* < 0.05) was achieved at concentrations of 19.53, 0.02, and 0.15 nM for TOP10/pCon-IND-C, TOP10/pCon-IND-C-B, and TOP10/pCon-IND-C-surB, respectively, yielding calculated LODs of 19.53, 0.04, and 0.08 nM.

As shown in Fig. 5, our progressive genetic optimization has significantly improved sensitivity. To provide context for our biosensing performance, we have included a comparative analysis with established analytical techniques in Table 3. While methods such as HPLC-ICP-MS achieve lower detection limits (0.003–0.028 nM), our platform's LOD of 0.04 nM is remarkably competitive considering its operational simplicity and cost-effectiveness. While individual sample detection requires longer incubation times than instrumental methods, the 96-well plate format allows for high-throughput parallel screening of numerous samples with minimal manual intervention. The low instrumental barrier and field-deployable nature make this platform especially suitable for large-scale preliminary screening and source tracking, acting as a practical complement to gold-standard instrumental techniques for analyzing trace MeHg in environmental monitoring.

**Table 3:** Comparison of analytical performance for MeHg detection between the developed biosensing platform and other reported methods^a^

Analytical technique	LOD (nM)	Linear range (nM)	Advantages	Limitations	Ref.
Whole-cell biosensor	0.04	0.02–1.22	Simple, low-cost, field-deployable	Longer detection time, cell culture required	This study
MSPE-HPLC-ICP-MS	0.003	23.2–23,191	High sensitivity, wide linear range	Expensive equipment, high operating costs	29
Online SPE-HPLC-ICP-MS	0.028	0.23–232	High automation, fast analysis	Complex system, high maintenance costs	30
Electrochemical sensor	0.23	4.6–116	Real-time monitoring, portable	Lower sensitivity	31
EC-CVG-AFS	0.009	1–24.9	Low detection limit	Cumbersome operation	32

a MSPE: magnetic solid-phase extraction; ICP-MS: inductively coupled plasma mass spectrometry; SPE: solid-phase extraction; EC-CVG-AFS: electrochemical cold vapor generation atomic fluorescence spectrometry.

Collectively, TOP10/pCon-IND-C-B, which features intracellular MeHg cleavage, achieved an optimal balance between sensitivity and a linear quantitative range. Therefore, this sensor was selected for subsequent evaluation of trace MeHg detection in complex environmental water samples.

### Assessment of TOP10/pCon-IND-C-B in diverse environmental water matrices

**Fig. 6:**
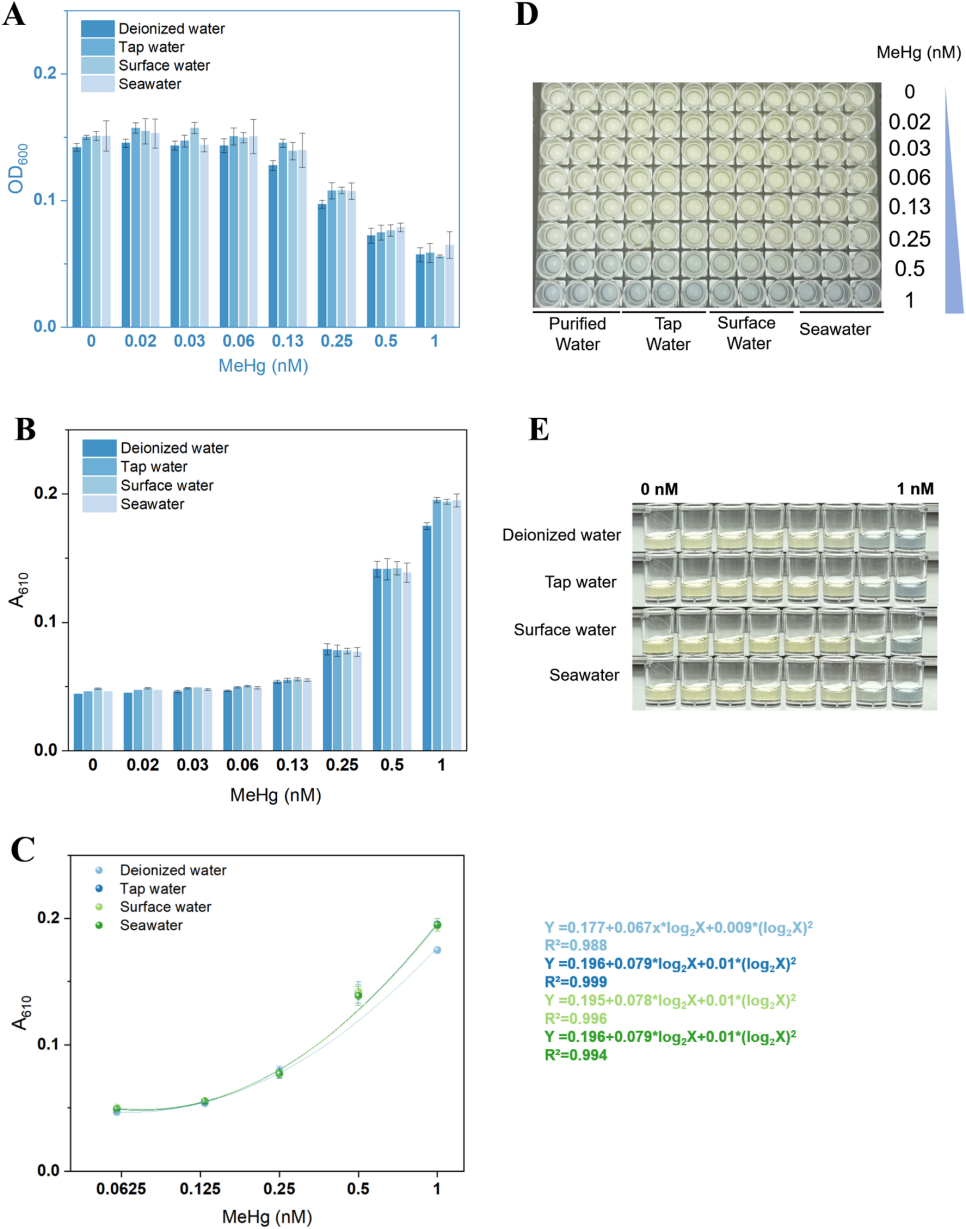
Validation of MeHg sensing in environmental water matrices. (A) Bacterial growth (OD_600_) after exposure to MeHg. (B) Pigment signal (*A*_610_) response. (C) Regression analysis of pigment signal *versus* MeHg concentration (log_2_ scale). (D) Representative images of bacterial cultures. (E) Representative images of culture supernatants.

Spiked MeHg was evaluated in deionized, tap, surface, and seawater samples using TOP10/pCon-IND-C-B. Sensor growth was comparable across all four matrices (Fig. 6A), with growth inhibition evident above 0.13 nM MeHg. Pigment signal increased significantly above this threshold (Fig. 6B).

Fig. 6C demonstrates that TOP10/pCon-IND-C-B maintained a consistent quantitative detection range (0.0625–1 nM) across all water matrices, including seawater, with highly overlapping response curves (*R*^2^ > 0.98). Dissolved organic matter in freshwater and high concentrations of inorganic ions in seawater did not significantly interfere with MeHg recognition, Hg(ii) transport, or indigoidine synthesis, indicating negligible matrix effects, which were also observed in whole-cell sensing of other toxic metals.^33,34^ Visually discernible blue color intensification was observed in all water samples with increasing MeHg concentration (Fig. 6D and E).

The selectivity of this biosensing platform is primarily determined by the high specificity of dimeric MerR for Hg(ii), which has been well documented for its ability to distinguish between other metal ions, such as Cd(ii), Pb(ii), Zn(ii), and Cu(ii).^8,18,19^ This molecular recognition specificity ensures minimal interference from common cations in environmental waters. Since MerB catalyzes the cleavage of the C–Hg bond in various organomercurials,^3^ our biosensor theoretically functions as a broad-spectrum detector for organomercury species beyond MeHg, including ethylmercury and phenylmercury. The primary determinant of response magnitude is the bioavailability of these organomercurials rather than MerB substrate specificity. Consequently, interference from dissolved organic matter or competing ligands is expected to be limited to their effect on MeHg uptake efficiency. In contrast, direct interference with the Hg(ii)-MerR binding event remains negligible. This inherent selectivity profile makes the platform particularly robust for screening mercury species in complex matrices.

While the WHO drinking water guideline for MeHg is set at 30 nM, the sub-nanomolar detection range of our biosensor (0.02–1.22 nM) is highly relevant for environmental monitoring because ambient MeHg concentrations in most natural water bodies are significantly lower than regulatory thresholds, and MeHg in uncontaminated or lightly contaminated waters is far below the 30 nM safety limit.^1,2^ Moreover, MeHg exhibits strong bioaccumulation and biomagnification, whereby even picomolar water concentrations can amplify through food webs to reach toxic levels in higher trophic organisms including humans, making monitoring far below safety limits essential for understanding exposure risks and implementing preventive measures.^35^ Sub-nanomolar sensitivity also enables early pollution detection and source tracking, allowing identification of emerging contamination sources before they escalate to hazardous levels, which is particularly crucial for industrial discharge monitoring and watershed protection.

Metabolic burden from indigoidine synthesis might impose a significant growth penalty, limiting the quantitative range.^21^ However, for high-concentration samples from pollution events, simple 1 : 10 or 1 : 100 dilution brings them into the linear range, an operation far simpler than the extraction/derivatization required by traditional methods and still compliant with green analytical chemistry principles.^36^ In contrast, an excessively wide dynamic range would sacrifice sensitivity and resolution at the low-concentration end, hindering early detection of trace contamination. We acknowledge that this range has limitations for compliance testing, but in most environmental monitoring scenarios, a narrow yet sensitive range is more valuable than a wide but insensitive one.

The observed trade-off between bacterial growth inhibition and pigment signal response is a common phenomenon in whole-cell biosensing, particularly when using fluorescent or chromoprotein reporters.^37,38^ In such systems, increasing analyte concentrations often simultaneously induce reporter synthesis and cellular toxicity, leading to signal attenuation at high doses. It typically necessitates calculation of normalized responses (*e.g.*, relative fluorescence units divided by OD_600_), which complicates data processing and introduces additional variability from dual-parameter measurements.^4^ In our indigoidine-based system, while both metabolic burden from pigment synthesis and MeHg cytotoxicity contribute to growth inhibition, the dose-response relationship remains quantitatively reliable within the ascending phase of the parabolic curve (0.02–1.22 nM). Within this sub-nanomolar range, the decrease in OD_600_ is minimal (<15%) and correlates linearly with MeHg concentration, allowing direct use of *A*_610_ values without normalization. This simplification enhances data analysis and lowers experimental uncertainty, strengthening the biosensing platform for field-deployable screening applications.

## Conclusion

We developed an enzyme-cascade-amplified whole-cell biosensor achieving sub-nanomolar methylmercury detection (LOD = 0.04 nM, linear range: 0.02–1.22 nM). TOP10/pCon-IND-C-B, featuring intracellular MerB cleavage, demonstrated robust performance across diverse water matrices (*R*^2^ > 0.98), including seawater, with negligible matrix effects. The water-soluble indigoidine pigment enabled direct naked-eye detection, offering a cost-effective alternative to instrumental methods. Notably, metabolic burden from pigment synthesis, rather than MeHg cytotoxicity, dominated the dose-response profile. This biosensor provides a potential platform for MeHg screening in environmental monitoring.

## Conflicts of interest

There are no conflicts to declare.

## Supplementary Material

RA-016-D5RA09313A-s001

## Data Availability

The data supporting this article have been included as part of the supplementary information (SI). Supplementary information: Table S1: DNA sequences of biological modules used in this study. Fig. S1: relationship between MeHg exposure dose and sensor cell growth. See DOI: https://doi.org/10.1039/d5ra09313a.
