# Supplementary material for: Enzyme-cascade-amplified colorimetric biosensing platform for sub-nanomolar methylmercury in environmental waters
Source: RSC Adv. 2026 Jan 7;16(3):2112–22. doi: 10.1039/d5ra09313a (PMC12777952; doi:10.1039/d5ra09313a)
Supplement: RA-016-D5RA09313A-s001 [file RA-016-D5RA09313A-s001.pdf]

Supplementary Information (SI) for RSC Advances.
This journal is © The Royal Society of Chemistry 2025

Table S1. DNA sequences of biological modules used in this study.

Vectors	DNA sequence	Description
pCon-IND	AGATCTCTAAGGCATAGCTGACCTTGCCAGGCCTGCTTCGCCCTGTAGTGACGCG<br>ATCAACGGGCAGGAAACATTCCCCTTTCGTGCATGGCAGGCGCACACGAGTTCAG<br>ACAGCACGGTTTCCATGCGCGCCAAGTCGGCCATCTTCTCGCGCACGTCCTTGAGC<br>TTGTGTTCGGCCAGGCTGCTGGCCTCCTCGCAGTGGGTGCCATCGTCGAGCCGCAA<br>CAGCTCGGCAATCTCGTCCAGACTGAACCCCAGCCGCTGTGCCGATTTCACGAATT<br>TCACCCGAACCACGTCCGCCTCCCCATAGCGGCGGATGCTGCCGTAAGGCTTGTCC<br>GGTTCCCGCAACAGGCCCTTGCGCTGATAGAAGCGGATTGTCTCCACGTTGACCCC<br>GGCCGCCTTGGCAAAAACGCCAATGGTCAGGTTTTCCAAATTATTTTCCATCGTTC<br>GCGCAACGCCTCCTGGTACCTGTGCTTTGTTAAAGGGTTTCAACAGATATTCAAAT<br>CGCTTGACTCCGTACATGAGTACGGAAGTAAGGTTACGCTATCCAATCCAAATTCA<br>AAAGGGCCAACGTTCTAGAAATAATTTTGTTTAACTTTAAGAAGGAGATATACAT<br>ATG	Hg(II) sensory module using a uncoupled circuit (*Xba*I-*Bgl*II-*Nde*I)
pCon-IND-C	GAGCTCTTGACAGCTAGCTCAGTCCTAGGTATAATGCTAGGttaaggaggtaaaaaaATGG<br>GTCTGATGACCCGTATCGCGGATAAAACCGGCGCGCTGGGTAGCGTTGTCAGCGC<br>GATGGGCTGCGCGGCGTGCTTCCCGGCGCTGGCGAGCTTCGGCGCCGCGATCGGC<br>CTGGGCTTCCTGAGCCAGTACGAAGGCCTGTTCATCAGCCGTCTGCTGCCGCTGTT<br>CGCGGCGCTGGCGTTCCTGGCGAACGCGCTGGGCTGGTTCAGCCACCGTCAGTGG<br>CTGCGTAGCCTGCTGGGTATGATCGGTCCGGCTATTGTTTTCGCAGCTACCGTTTG<br>GCTGTTAGGTAACTGGTGGACCGCTAACCTGATGTATGTTGGTTTAGCTCTGATGA<br>TTGGTGTTAGCATTTGGGATTTTGTTTCTCCGGCACACCGTCGTTGCGGTCCGGATG<br>GCTGCGAACTGCCGGCTAAACGTCTGtaaAAGCTT	Hg(II) transport module MerC (*Sac*I-*Hind*III)
pCon-IND-C-B	AAGCTTTCGAGATTACACTTTATGCTTCCGGCTCGTATAATGTGTGGattaaagaggagaaa<br>atgAAACTGGCGCCGTATATCCTGGAACGTCTGACCAGCGTTAATCGTACCAACGGT<br>ACCGCGGATCTGCTGGTTCCGCTGCTGCGTGAACTGGCGAAAGGTCGTCCGGTTTC<br>TCGTACCACCCTGGCGGGTATCCTGGATTGGCCGGCGGAACGTGTAGCCGCGGTCC<br>TGGAACAGGCGACCTCTACCGAATACGACAAAGACGGTAACATCATCGGTTATGG<br>CCTGACGCTGCGTGAAACCTCCTACGTTTTCGAGATCGACGATCGTCGCCTGTACG<br>CGTGGTGCGCGCTGGACACCCTGATCTTCCCGGCGCTGATCGGTCGTACCGCGCGT<br>GTTAGCAGCCACTGCGCCGCGACCGGCGCGCCGGTTAGCCTGACCGTTTCACCGTC<br>TGAAATCCAGGCGGTGGAGCCGGCAGGCATGGCGGTTAGCCTGGTGCTGCCGCAG<br>GAAGCGGCGGATGTTCGTCAGTCTTTCTGCTGCCACGTTCACTTCTTCGCGTCTGTT<br>CCGACCGCGGAAGATTGGGCGAGCAAGCACCAGGGTCTGGAAGGGCTGGCGATC<br>GTGAGCGTTCACGAAGCGTTCGGCCTGGGCCAGGAATTCAACCGTCACCTGCTGC<br>AGACCATGAGCAGCCGCACCCCG taaCTCGAG	Organic Hg lyase module (*Hind*III-*Xho*I)
pCon-IND-C-surB	GAGCTCTCGAGATTACACTTTATGCTTCCGGCTCGTATAATGTGTGGattaaagaggagaaa<br>atgAACGATGATAAAGTTCTGGTTCTGCGTACCTGCGCGAACAACATGGCGGATCAC<br>TGCGGCCAGATCTGGCCGGTTAGCGGCGTTGTAGAATGCAAATACTGGGAACCGA<br>CCCGTAAACTGGAAAACGGCCTGGCGGGCCTGCTGTGGGGCAAAGGCGCGAGCAC<br>CCACCTGAACATGCAAGCTGACGCCCGCTGGGTGATCTGTGAAGTAGCAGTGTCT<br>GACATTATCTTCCTGGACGCCCAGGGTGGCGTGAAGTTCCCGCGCGCTGAAGTGGT<br>TCACGTAGGCACCCGTAACAGCGCGGCGGGTTACATCAGCGCGAACATCGCGAGC<br>TACGCGAGCAGCACCGTTGCGCTGAACGAAACCTTCGTTTTCCCGGAAGTTCGTAC<br>CGAAACCAAAGTTGACTTCCCGGCGAGCCCGGCGACCGCGGATAGCACCTTCGAT<br>ATTGATCGTCACGCGACCATCCAGGGCCCGCAGACCCTGGAAACCGCGGTTGCGG<br>ATCCgATGAAACTGGCGCCGTATATCCTGGAACGTCTGACCAGCGTTAATCGTACC<br>AACGGTACCGCGGATCTGCTGGTTCCGCTGCTGCGTGAACTGGCGAAAGGTCGTCC<br>GGTTTCTCGTACCACCCTGGCGGGTATCCTGGATTGGCCGGCGGAACGTGTAGCCG<br>CGGTCCTGGAACAGGCGACCTCTACCGAATACGACAAAGACGGTAACATCATCGG<br>TTATGGCCTGACGCTGCGTGAAACCTCCTACGTTTTCGAGATCGACGATCGTCGCC<br>TGTACGCGTGGTGCGCGCTGGACACCCTGATCTTCCCGGCGCTGATCGGTCGTACC<br>GCGCGTGTTAGCAGCCACTGCGCCGCGACCGGCGCGCCGGTTAGCCTGACCGTTTC<br>ACCGTCTGAAATCCAGGCGGTGGAGCCGGCAGGCATGGCGGTTAGCCTGGTGCTG<br>CCGCAGGAAGCGGCGGATGTTCGTCAGTCTTTCTGCTGCCACGTTCACTTCTTCGC<br>GTCTGTTCCGACCGCGGAAGATTGGGCGAGCAAGCACCAGGGTCTGGAAGGGCTG<br>GCGATCGTGAGCGTTCACGAAGCGTTCGGCCTGGGCCAGGAATTCAACCGTCACC<br>TGCTGCAGACCATGAGCAGCCGCACCCCGtaaCTCGAG	Surface displayed organic Hg lyase module (*Hind*III-*Xho*I)
IdgS-Sfp module	catATGACCCTGCAGGAAACCTCCGTTCTGGAACCGACCCTGCGTGGCACCACTACCCTGCCGGA<br>CCTGCTGGCGAAACGTGTAGCAGAACATCCAGAAGCAACCGCAGTGGCTTACCGCGACGAAAA<br>ACTGACCTACCGTGAACTGGCGAGCCGTTCTAGCGCACTGGCGGAATACCTGCGCCACCTGGGT<br>GTTTCTACCGATGACTGCGTTGGTCTGTTTGTAGAACCGAGCATCGATCTGATGGTAGGCGCTTG<br>GGGCATCCTGTCTGCTGGTGCAGCCTACCTGCCGCTGAGCCCAGAATACCCGGAAGATCGCCTG<br>CGCTATATGATCGAGAACAGCCAGGCAAAAATCATCCTGGCTCAGCAACGCCTGGTGACCCGTC<br>TGCGTGAACTGGCCCCGCAGGATGTACGCGTGGTGACCCTGCGTGAATCCGAAGCATTCGTACT<br>GCCAGAAGGTCAGGTTGCGCCGGCAATTGAAGGTGCGCGCCCGGACAGCCTCGCGTACGTGATT<br>TACACCTCTGGCTCCACCGGTAAGCCGAAAGGCGTAATGATTGAACACCACTCGATCGTTAGTC<br>AGCTGGGGTGGCTGCGTGAAACCTACGGCATTGACCGTAGCAAAACCATTCTGCAGAAAACCCC<br>GATGTCTTTTGACGCTGCCCAGTGGGAAATTCTGAGCCCGGCTAACGGCGCCACCGTCGTGATG<br>GGGGCCCCAGGTGTTTACGCGGACCCGGAAGGTCTGATTGAAACCATCGTGAAATATGGCGTCA<br>CCACGCTGCAGTGCGTTCCGACGCTGCTGCAGGGCTTGCTGGATACTGAAAAATTCCCGGAATG<br>CACGAGCCTGCAGCAGATCTTTAGCGGCGGTGAAGCTCTGAGCCGTCTGTTGGCTATTCAGACC<br>ACCCAGGAAATGCCGGGTCGTGCGCTGATTAATGTCTACGGCCCGACTGAATGTACCATTAACT<br>CCTCTTCTTACGCAGTTGACCCGGCTGAACTGGGCGAAGCTCCACAATCTATCTCTATCGGCGCG<br>CCGGTGGCTGATACTGAATACCATATCCTGGGCAAAGAGGATCTGAAACCGGTGGGTGTAGGTG<br>AAATTGGCGAACTCTACATTGGTGGCGGCCAGTTGGCGCGTGGTTATCTGCATCGTCCGGATCTG<br>ACCGCTGAACGTTTCCTGGAAATCGAAGTGACCGAAGGTGCGGGCCCGGTTCGCCTGTACAAGA<br>CGGGTGACCTGGGTCAGTGGAACCCGGACGGCACCGTGCAGTTTGCGGGCCGTGCAGATAATCA<br>AGTCAAATTGCGTGGCTACCGTGTTGAGTTAGATGAAATTTCACTGGCGATTGAGAACCATGAT<br>TGGGTCCGTAACGCGGCTGTTATCGTAAAAAACGATGGTCGTACCGGTTTCCAGAACCTGATTG<br>CTTGCGTAGAACTGAGCGAAAAAGAAGCCGCGCTGATGGATCAGGGTAACCATGGTAGCCACC<br>ATGCATCTAAAAAATCGAAACTGCAAGTTAAAGCACAGCTGTCTAATCCTGGCCTGCGTGACGA	Indigoidine reporter module (*Nde*I-*Sac*I)

	TGCGGATTTAGCGGCACGTGTTGCCTACGACCTGCCTGGCGCGGAACCGACCCCGGAACAGCGT<br>TCGCGTGTTTTTGCCCGTAAAACCTACCGTTTTTACGAAGGTGGTGCTGTCACCGAAGCTGATCT<br>GCTGGCGCTGCTGGGCGGCCAGGTCCCGGCGGCATACAGCCGTAAAGCCGCGGATCTGGCTCCT<br>GCTGAACTGGGACAAATCTTGCGTTGGTTTGGTCAGTACCTGTCTGAGGAACGTCTGTTACCGAA<br>ATATGGCTACGCGAGCCCAGGAGCGTTGTATGCGACCCAGTTGTACTTTGAACTGGAAGGTGTT<br>GGTGGTCTGCAGCCGGGTTACTACTACTACCAGCCGCAGCGTCACCAGCTGGTTTTAATCTCTGA<br>AAAAGCGGCGACCGGGCGTCCGACGGCTCACATCCATTTCATCGGTAAACGTGGTGGCATTGAA<br>CCGGTGTACAAAAACAATATCCAAGAGGTTCTGGAGATTGAAACCGGCCACATAGTAGGCTTAT<br>TTGAACAGGTACTGCCGGCCTATGGCCTGGATATCCGTGATCTGGCGTATGAACCGGCCGTTCGT<br>GATTTACTGGATGTTCCGGAAGAAGATTTCTACCTGGGTACCTTCGAACTGGTTCCGCACACGGG<br>CCGTCGTGAAGATCACGCGGAAGTGTACGTGCAGACCCATGGCTCTAAAGTGGCTAACCTGCCG<br>GAAGGTCAGTATCGTTATGCAGACGGCACCCTGACCCGTTTCAGCGATGACATTGTACTGAAAA<br>AACAGGTCATCGCGATTAATCAGTCCGTTTATCAGGCAGCCTCGTTCGGAATCTCCGTTATTAGT<br>CGTGCACCGGAAGAATGGATGCATTACGTCACCCTGGGTAAAAAACTGCAGCACCTTATGATGA<br>ACGGCCTGGGTCTGGGCTTCATGTCGTCCGGTTACAGCTCTAAAACCGGTAACCCGCTGCCGGC<br>ATCCCGTCGTATTGATTCTGTCCTGCAGGCGAATGGTGTAGAAAGCGGCCCGTCTTATTTCTTCG<br>TAGGTGGTCGTGTTAGTGATGAACAGTTGGGCCATGAGGGTATGCGTGAAGATAGCGTTCACAT<br>GCGTGGCCCGGCGGAACTGATTCGCGATGACCTGGTGTCTTTTCTTCCGGACTATATGATTCCGA<br>ACCGCGTAGTAGTCTTTGAACGTCTGCCGCTGTCCGCTAACGGTAAAATTGACGCCAAAGCCCT<br>CGCGGCAAGCGATCAGGTAAACGCGGAACTGGTTGAACGTCCGTTCGTGGCTCCGCGTACTGAA<br>ACAGAAAAAGAAATCGCAGAAGTTTGGGCTAAATCCCTGCGTCGCGAGTCTGTTTCAGTTCAAG<br>ATGATTTCTTCGAAAGCGGTGGTAACAGCCTGATCGCGGTTGGCCTGATTCGTGAACTGAACAG<br>CCGCCTGGGTGTTAGCCTGCCGCTGCAGAGCGTTCTGGAGTCCCCGACTGTTGAAAAATTAAGC<br>CGCCGTCTGGAACGCGAGGTGGCCCAGGAAAGCAGCCGCCTGGTGCGTCTGCATGCTGAAACTG<br>GTAAAGATCGTCCGGTTCTGTGTTGGCCGGGCCTGGGCGGCTACCCGATGAACCTGCGCACCCT<br>GGCAGGCGAAATCGGCCTGGGTCGTTCCTTCTACGGTATCCAGGCACACGGCATCAACGAAGGT<br>GAAGCACCGTATGCGACCATCACTGAAATGGCGAAAGCGGATATTGAAGCAATCAAAGAACTG<br>CAGCCTAAAGGCCCGTATACCCTGTGGGGTTATTCATTCGGCGCTCGCGTTGCTTTCGAAACCGC<br>GTATCAGCTGGAACAGGCTGGTGAGAAAGTGGATAACTTGTTCCTTATTGCGCCTGGTTCCCCG<br>ACCGTTCGCGCGGAAAACGGTAAAGTTTACGGCCGCGAGGCGAGCTTCGCTAACCGCGCTTATA<br>CTACCATCCTGTTCTCTGTATTCACGGGTACGATCTCCGGTCCTGATCTGGAAAAATGCCTGGAA<br>TCAGCTACTGACGAAGAATCGTTTGCGGGTTTCATTAGCGAACTGAAAGGCATCGATGTTGACC<br>TGGCTAAACGCATCATTTCCGTAGTGGGTCAGACCTACGAATTTGAATACTCCTTCCGTGAACTG<br>GCTGAACGCACCCTGGCCGCACCGGTGACCATTTTCAAAGCACGTGGTGACGACTACAGCTTTA<br>TCGAAAACTCTAACGGCTACAGCGCTGAACCGCCGACTGTGATCGATCTGGACGCGGATCACTA<br>CTCTCTGCTGCGCACCCCGGATATCGGTGAACTGGTTAAACACATCCGTTATCTGCTGGGTGAGta<br>attaaggaggtaaaaaaaatgAAAATCTATGGTATCTACATGGATCGTCCGCTGAGCCAGGAAGAGAATGAG<br>CGCTTCATGACGTTTATCTCTCCGGAGAAACGTGAAAAATGTCGCCGCTTCTATCATAAAGAAG<br>ATGCTCATCGTACCCTGCTGGGCGATGTTCTGGTTCGTTCTGTTATCTCTCGTCAGTACCAGCTGG<br>ATAAAAGCGATATCCGTTTCTCAACCCAGGAATACGGTAAACCGTGCATCCCGGATCTGCCGGA<br>TGCGCACTTCAACATCAGCCACAGCGGCCGTTGGGTTATCGGTGCGTTCGATTCTCAGCCGATCG<br>GTATCGATATCGAAAAAACCAAACCGATCAGCCTGGAAATCGCGAAACGTTTCTTCTCTAAAAC<br>CGAATACTCTGATCTGCTGGCGAAAGATAAAGATGAACAGACCGATTACTTCTACCACCTGTGG<br>AGCATGAAAGAATCTTTCATCAAACAGGAAGGCAAAGGTCTGAGCCTGCCGCTGGATAGCTTCT<br>CTGTTCGTCTGCACCAGGATGGTCAGGTTAGCATCGAACTGCCGGATAGCCACAGCCCGTGCTA<br>CATCAAAACCTACGAAGTTGATCCGGGTTACAAAATGGCGGTTTGCGCGGCGCACCCGGATTTC<br>CCGGAAGATATCACCATGGTTAGCTACGAAGAACTGCTGtaaGAGCTC	

The MerR coding sequence is shown in green. The Hg(II)-responsive divergent Promoter P_{mer} is shown in a pink background. The constitutive promoters (uppercase) and ribosome sites (lowercase) are all shown in a grey background. The Hg(II) transport module MerC-encoding sequence is shown in grey-red. The MerB-encoding sequence is shown in light-blue. The outer membrane anchoring protein InaPb-encoding sequence is shown in orange. The start codon is shown in a green background. The end codon is shown in a red background. Restriction sites are underlined.

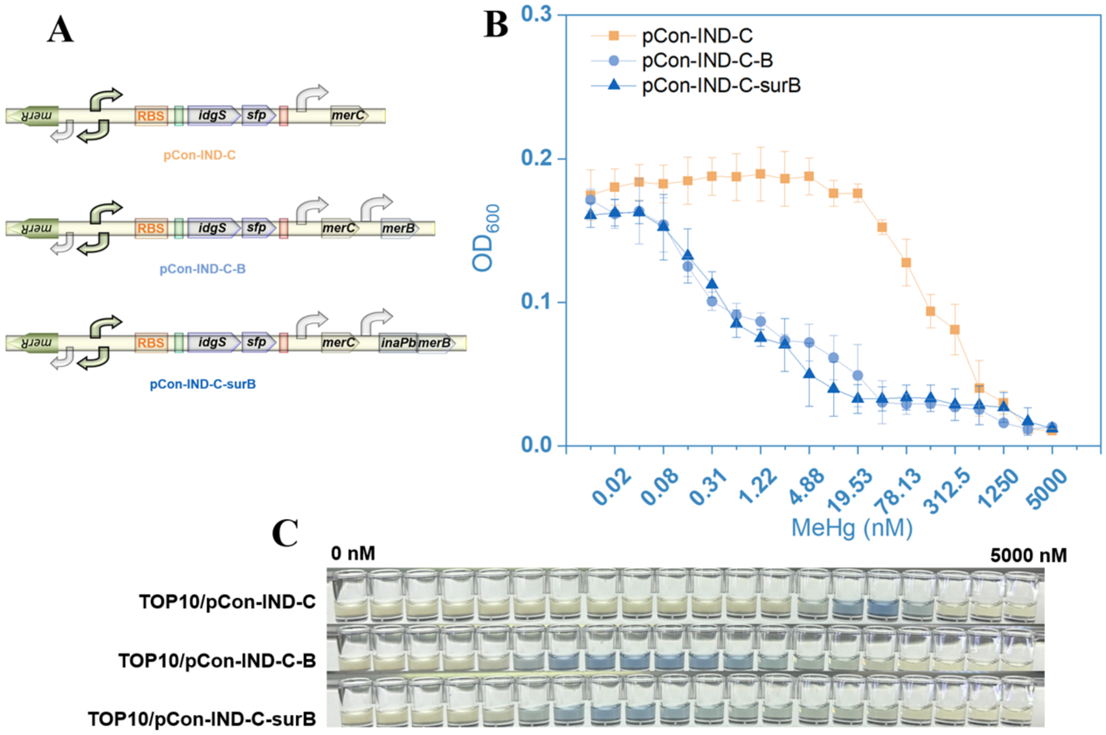

Figure S1. Relationship between MeHg exposure dose and sensor cell growth. (A) Schematic diagram of the genetic construction of the three sensor plasmids. (B) Bacterial growth of the sensor series after exposure to elevated concentrations of MeHg, represented by changes in cell density. (C) Comparison of bacterial cultures of the three biosensors exposed to increasing MeHg concentrations.
